# The Dental Health of primary school children living in fluoridated, pre-fluoridated and non-fluoridated communities in New South Wales, Australia

**DOI:** 10.1186/1472-6831-15-9

**Published:** 2015-01-21

**Authors:** Anthony S Blinkhorn, Roy Byun, George Johnson, Pathik Metha, Meredith Kay, Peter Lewis

**Affiliations:** Faculty of Dentistry, University of Sydney, 1 Mons Road, Westmead, NSW 2145 Australia; South Western Sydney Local Health District, Liverpool, NSW 2170 Australia; Faculty of Medicine, University of Sydney, Sydney, NSW 2000 Australia; Breast Screen NSW, North Sydney Local Health District, Royal North Shore Hospital, Sydney, NSW 2065 Australia; Central Coast Local Health District, Ourimbah, NSW 2258 Australia

**Keywords:** Water fluoridation, Dental caries, Children, Australia

## Abstract

**Background:**

The Local Government Area of Gosford implemented a water fluoridation scheme in 2008. Therefore the opportunity was taken to record the dental health of primary school children aged 5–7 years prior to the fluoridation and compare the results with other communities in NSW with different access to fluoridated water. The aim was to compare the oral health of New South Wales (Australia)s 5–7 year olds living in fluoridated, and non- fluoridated communities. One of the areas was due to implement water fluoridation and is termed the pre-fluoridation site.

**Methods:**

Pupils in the first year of Public and Catholic Schools in three areas of NSW were recruited. Class lists were used to draw a sample of approximately 900 per area. This number allowed for a non-response rate of up to 30 per cent and would give a sample sufficient numbers to allow statistical inferences to be drawn. Children whose parents consented received a dental examination and the clinical data was collected on mark sense cards.

**Results:**

In the 3 areas the proportion of children who received a dental examination varied; 77.5% (n = 825) for the fluoridated area, 80.1% (n = 781) for the pre-fluoridated area and 55.3% (n = 523) for the non-fluoridated area. The mean dmft was 1.40 for the fluoridated area, 2.02 for the pre-fluoridated area and 2.09 for the non-fluoridated area. These differences were statistically significant (p < 0.01). Differences were also noted in the proportion of children who were caries free, 62.6% fluoridated area, 50.8% for the pre-fluoride area and 48.6% for the non-fluoride location.

**Conclusion:**

The children living in the well-established fluoridated area had less dental caries and a higher proportion free from disease when compared with the other two areas which were not fluoridated. Fluoridation demonstrated a clear benefit in terms of better oral health for young children.

## Background

The State of New South Wales in Australia has a long history of water fluoridation in common with many other countries throughout the world [1, 2]. In the 1950’s levels of dental caries in NSW children were amongst the highest in the world. The mean Decayed, Missing and Filled Teeth score (DMFT) for 12 year olds at that time was 9.0; a huge burden of disease and treatment need [3] In an effort to improve the oral health of people living in NSW fluoridation of public water supplies was given a high priority. The first water fluoridation scheme in NSW was opened in Yass in 1956 followed by Tamworth in 1963 and the City of Sydney. By June 2010 approximately ninety four per cent of the NSW population had access to fluoridated water [4, 5] and epidemiological surveys have charted a dramatic decline in the prevalence of dental caries in NSW over the last 50 years [6–8]. However the addition of new communities having fluoridated water has slowed. In an effort to offer all NSW residents access to fluoridated water the NSW Department of Health agreed in 2007 to fund 100 per cent of the capital costs of installing any new fluoridation plant. This investment is an economically sound policy [9] as the treatment of dental caries requires a substantial financial allocation from State and Federal funded health budgets, as well as considerable finance from individuals and private health insurance companies. The great advantage of water fluoridation over other forms of fluoride delivery is that it does not require any effort on the part of the target population, as water consumption is a universal behaviour. Therefore water fluoridation should be the preventative measure of choice, as it will decrease oral health inequalities, because compliance with water fluoridation is automatic, requiring no behavioural change [10].

It has been shown that children from NSW living in areas without water fluoridation have higher caries rates than their peers in fluoridated areas [11]. However these data were gathered from a sample of Public Dental Service patients, whereas the majority of child dental care in NSW is provided by private dental practitioners. Thus the results do not reflect a representative picture of oral health children living in NSW. The opportunity to undertake a more rigorous evaluation of fluoridation was presented when the Local Government Area (LGA) of Gosford voted to implement a fluoridation scheme in 2008. NSW Health (Centre for Oral Health Strategy) in collaboration with the Australian Dental Association (NSW Branch) funded a research program in Gosford LGA to record the dental health of children, pre fluoridation then assess the impact of fluoridation on children’s dental health over the next five years. The first aim of the research was to compare the prevalence of dental caries of 5–7 year old children in Gosford LGA prior to fluoridation with a well-established fluoridation scheme and a location which currently has no plans to implement fluoridation.

## Methods

The City of Gosford LGA on the Central Coast is the largest area in NSW to have agreed to implement a new fluoridation scheme in recent years, and had an estimated resident population of 162,017 in 2006. The study design included a comparator LGA, Wyong Shire, which has been fluoridated for over 40 years and it is also situated on the Central Coast of NSW with an estimated resident population of 142,724 in 2006. In 2006, the population of Wyong LGA was slightly younger than Gosford LGA (median age 39 vs 40), had a lower median household income (median $770 vs $944) and a lower Index of Relative Socio-economic Advantage and Disadvantage score (955 vs 1013).

A non-fluoridated comparison site is more difficult to identify, given the widespread coverage of water fluoridation schemes in NSW. The sample size calculation suggested a sample size of 500 per group to detect a difference in mean dmft in 5 year olds of 0.3 between the fluoridated and un-fluoridated groups with a power of 0.8 at a significance level of 0.05. Most of the un-fluoridated communities are smaller than Gosford and Wyong and more rural, so two control areas were required. Inevitably there will be some social differences. The non-fluoridated comparator sites were situated in the North Coast of NSW and were the coastal LGAs of Ballina and Byron Shires, which had estimated resident populations of 40,266 and 30,635, respectively, in 2006 [12].

The sampling units were all the 27 State and Catholic Schools in Gosford LGA, 22 in Wyong LGA, and 39 in Ballina and Byron LGAs. The schools were drawn at random from master school lists until the individual school roles added up to approximately 900 per area. This number allowed for a response rate of approximately 70 per cent. A screening study on ocular health in the same geographical locations achieved an 80–85 per cent positive consent rate [13], however we were concerned that monitoring oral health to assess the potential benefits of fluoridation might not achieve similar high recruitment success. The children were examined at the school utilising portable equipment, including a dental light, a Mini compressor to provide air to dry the teeth and special dental chairs. Where possible, two calibrated Dental examiners visited each school to reduce the impact on children’s lessons. The examination procedure and diagnostic system were the same as that used in the 2007 NSW Child Dental Health Survey [14] where individual tooth surfaces were classified as decayed, missing because of caries, or filled because of caries. One of the organisers of the 2007 Child Dental Health Survey undertook the examiner training, and was the designated Gold Standard.

The diagnostic system was based on a visual examination of an air dried tooth. The diagnosis of dental caries was only assigned if there was a visual break in the enamel surface or marked shadowing. All examiners had been trained for the 2007 NSW Child Dental Health Survey, but revision sessions were arranged at a central site to maintain examiner consistency, using an experienced gold standard examiner (ASB). Differences in diagnosis were discussed and the need for consistency reinforced. Ten per cent of the children were re-examined to record intra-examiner consistency, and the gold standard examiner undertook five joint dental inspections with each of the six study examiners.

Data were collected on mark sense cards and scanned utilising Tele-Form Software [15], which uses optical character recognition to generate a useable data file.

Data was then exported to Microsoft Access and Microsoft Excel for cleaning and checking. Caries experience was measured using the dmft index (deciduous dentition), which is the number of teeth that are decayed, missing or filled due to caries. In addition the proportions of children caries free and the significant caries index (SiC) [16] were also recorded. Univariate and multivariate logistic regression (adjusted odds ratios) and negative binomial regression analyses (incidence rate ratios) were used to identify significant independent associations between risk factors with caries experience and dmft counts. Significance was p-value of less than 0.05. Data were analysed using SAS version 9.2.

The study was approved by the State Education Research Process (SERAP) of the NSW Department of Education and Training. The Catholic Education Commission also gave permission to involve schools within their jurisdiction. (SERAP number 2008052) The South West Area Health Service (SWAHS) Human Research Ethics Committee granted ethical approval for the school based surveys; HEREC 2008 / 314.18 (2758); All RED 08 / WMEAD/ 57.

## Results

All the schools invited to participate in the research in the Wyong LGA (18 schools) and Gosford LGA (16 schools) gave a positive response, giving eligible populations of 1,065 and 932 respectively. The schools in Ballina and Byron LGAs are smaller and 27 schools were needed to give an overall population of around 900 children. Four school principals refused to join the study, expressing concerns that parents would be worried about water fluoridation research, as the use of fluoride had attracted considerable negative publicity. These were replaced by four other schools drawn at random from the school list for Ballina and Byron LGAs and a total of 945 consent forms were sent to the schools. In the fluoridated area (Wyong LGA), 825 (77.5%) received dental examinations, the figures for the pre-fluoridated area (Gosford LGA) were, 781 (80.1%) examined and for the non-fluoridated area (Ballina and Byron LGAs), 523 (55.3%) examined.

Six dental examiners were seconded to the study by the participating Area Health Services. Each examiner had a dental assistant to act as a scribe and liaise with the school office.

The number of examiners per school varied according to their Public Service clinical timetable. Four examiners undertook inspections in Wyong and Gosford whilst two examiners were available in North Coast. Each examiner completed five joint examinations with the gold examiner and reasonable intra- class correlations of 0.86 -0.97 for dmft scores were noted. When their own clinical data were compared there were acceptable levels of agreement for missing, decayed or filled teeth. Intra-class correlations (ICC) ranged from +0.79 to 0.91. ICC values range from a negative – 1.0 to a maximum of 1.0 with higher values representing greater agreement [17].

A description of the population by demographic characteristics is shown in Table 1. It can be seen that the three groups (Table 1) of participants were broadly similar in most aspects. However the fluoridated area had a slightly higher but significant (p = 0.04) proportion (43.9%) of individuals whose parents were concession card holders when compared to the pre-fluoridated area (38.0%) and non-fluoridated area (39.2%). In addition, the fluoridated area had a slightly higher proportion of Aboriginal children (7.1%) than in the pre-fluoridated area (5.0%) and non-fluoridated area (4.4%), although this was not significant (p = 0.07). None of the other differences between groups for any of the social characteristics recorded were statistically significant.

**Table 1:** **Characteristics of survey participants**

	Fluoridation status			Total
Characteristic	Fluoridated	Pre-fluoridated	Non-fluoridated	
	n (%)	n (%)	n (%)	n (%)
**Gender**				
Male	419 (50.7)	380 (48.7)	263 (50.2)	1,062 (49.9)
Female	406 (49.3)	401 (51.31)	260 (49.8)	1,067 (50.1)
**Age**				
5	345 (41.8)	360 (46.1)	115 (22.0)	820 (38.5)
6	475 (57.6)	416 (53.3)	374 (71.5)	1,265 (59.4)
7	5 (0.6)	5 (0.6)	34 (6.5)	44 (2.1)
**Concession cardholder**				
Yes	362 (43.9)	297 (38.0)	205 (39.2)	864 (40.6)
No	463 (56.1)	484 (62.0)	318 (60.8)	1265 (59.4)
**Aboriginal and/or Torres Strait Islander**				
Yes	58 (7.1)	39 (5.0)	23 (4.4)	120 (5.6)
No	695 (84.2)	660 (84.5)	468 (89.5)	1,823 (85.6)
Not stated	72 (8.7)	82 (10.5)	32 (6.1)	186 (8.8)
**Maternal country of birth**				
English speaking background	776 (94.0)	708 (90.6)	479 (91.6)	1963 (92.2)
(*Australia*)	(*723*) (87.6)	(*656*) (84.0)	(*426*) (81.4)	(*1,805*) (84.8)
Non-English speaking background	41 (5.0)	65 (8.3)	41 (7.8)	147 (7.0)
Not stated	8 (1.0)	8 (1.1)	3 (0.6)	19 (0.8)

The mean dmft scores and the proportions caries free are detailed in Table 2. It can be seen that the un-fluoridated and the pre-fluoridated areas had similar mean dmft scores of 2.09 and 2.02, whilst the established fluoridated area had a significantly lower mean dmft score of 1.40 (p < 0.01). Of particular note is the considerable difference in the proportions of caries free children according to location, fluoridated area 62.6%, pre-fluoridated area 50.8% and 48.6% for the un-fluoridated area (Table 2). These differences were statistically significant (p < 0.01). There were marked differences with the dmft and proportions caries free according to Aboriginal status (Table 2). Significant differences were noted in the mean dmft of 1.70 among non-Aboriginal children compared with a mean dmft of 2.80 for Aboriginal children (p < 0.01). Likewise, the proportions free from caries were 56.8% for non-Aboriginal children and 37.5% for Aboriginal children (p < 0.01). The SiC records the dmft of the 30 per cent of children with high levels of caries. Table 2 shows that the mean SiC scores were higher for all areas, but the fluoridated area had a lower score of 4.42 when compared with pre-fluoridated (5.85) and non-fluoridated (5.97) areas. Table 3 indicates that the fluoridated area had mean score of ‘d’ = 0.92 whereas the other two areas had mean ‘d’ scores of 1.42 for the pre-fluoridated area and 1.45 for the non-fluoridated area. Table 4 presents multi-variate analysis of dmft and caries experience, and shows that after controlling for various factors, the positive impact of water fluoridation on oral health, with children in the pre-fluoridated and non-fluoridated areas experiencing on average 38.0% and 53.0% higher mean dmft scores than children in the fluoridated area. Aboriginality and cardholder status were associated with poorer oral health.

**Table 2:** **Oral health indicators, dmft, caries free percent and significant caries index**

Variables	dmft		Caries free		SiC	
	Mean	95% CI	%	95% CI	Mean	95% CI
**Fluoridation status**						
Fluoridated	1.40^#^	1.22-1.58	62.6^#^	59.2-65.9	4.42	4.04-4.81
Pre- fluoridated	2.02**	1.80-2.23	50.8**	47.3-54.3	5.85	5.47-6.22
Non-fluoridated	2.09**	1.84-2.35	48.6**	44.3-52.9	5.97	5.58-6.37
**Indigenous status**						
Non-Aboriginal	1.70^#^	1.57-1.83	56.8^#^	54.6-59.1	5.17	4.92-5.41
Aboriginal	2.80**	2.16-3.44	37.5**	28.8-46.2	7.25	6.14-8.36
**Cardholder status**						
Non-cardholder	1.42^#^	1.28-1.56	61.0^#^	58.3-63.7	4.43	4.15-4.71
Cardholder	2.35**	2.13-2.57	45.7**	42.4-49.0	6.56	6.20-6.91
**Mother’s country of birth**						
English speaking	1.76^#^	1.63-1.89	55.1^#^	52.9-57.3	5.27	5.03-5.51
Non-English speaking	2.37 ^NS^	1.85-2.89	50.3 ^NS^	42.3-58.4	6.67	6.01-7.31
**Gender**						
Male	1.86^#^	1.69-2.03	53.9^#^	50.9-56.9	5.47	5.16-5.78
Female	1.74 ^NS^	1.56-1.91	55.8 ^NS^	52.8-58.7	5.25	4.92-5.59
**Age**						
5	1.74^#^	1.54-1.94	55.6^#^	52.2-59.0	5.23	4.83-5.63
6	1.82 ^NS^	1.66-1.97	54.8 ^NS^	52.0-57.5	5.41	5.12-5.69
7	2.41 ^NS^	1.44-3.38	40.9 ^NS^	26.4-55.4	6.50	5.04-7.96
**Total**	**1.80**	**1.68-1.92**	**54.8**	**52.7-56.9**	**5.37**	**5.14-5.60**

^#^referent, *p < 0.05; **p < 0.01; NS - not significant.

**Table 3:** **dmft components by fluoridation status**

Fluoridation status	Decayed		Missing		Filled		dmft		d/dmft
	Mean	SE	Mean	SE	Mean	SE	Mean	SE	(%)
**Fluoridated**	0.92	0.07	0.20	0.03	0.29	0.04	1.40	0.09	65.2
**Pre- fluoridated**	1.42	0.08	0.19	0.03	0.41	0.04	2.02	0.11	69.4
**Non-fluoridated**	1.45	0.10	0.06	0.02	0.58	0.06	2.09	0.13	70.5
**Total**	**1.23**	**0.05**	**0.16**	**0.02**	**0.40**	**0.03**	**1.80**	**0.06**	**68.6**

**Table 4:** **Multivariate analysis of dmft and caries experience**

Variables	dmft			Caries experience		
	IRR	95% CI	P	OR	95% CI	P
**Fluoridation status**						
Fluoridated	1.00^#^			1.00^#^		
Pre-fluoridated	1.38	1.14-1.67	<0.01	1.62	1.31-2.01	<0.01
Non-fluoridated	1.53	1.23-1.89	<0.01	1.86	1.46-2.37	<0.01
**Indigenous status**						
Non-Aboriginal	1.00^#^			1.00^#^		
Aboriginal	1.45	1.03-2.03	0.03	1.94	1.31-2.88	<0.01
**Cardholder status**						
Non-cardholder	1.00^#^			1.00^#^		
Cardholder	1.69	1.43-2.00	<0.01	1.86	1.54-2.25	<0.01
**Mother’s country of birth**						
English speaking	1.00^#^			1.00^#^		
Non-English speaking	1.41	1.02-1.93	0.04	1.13	0.79-1.62	0.51
**Gender**						
Male	1.00^#^			1.00^#^		
Female	0.91	0.77-1.07	0.25	0.89	0.74-1.07	0.20
**Age**						
5	1.00^#^			1.00^#^		
6	0.99	0.83-1.19	0.95	0.99	0.82-1.20	0.92
7	1.26	0.72-2.21	0.42	1.65	0.86-3.17	0.13

^#^referent, IRR – incidence rate ratio; OR – odds ratio.

## Discussion

In 2007 New South Wales Health established a program of regular population based epidemiological dental surveys which used random selection to investigate and report on the Oral Health of Children and Adolescents. The first survey was The Child Dental Health Survey undertaken in 2007 [18] and this was followed in 2010 by the Teen Survey recording the oral health of 14–15 year olds [19]. A follow up Child Survey is currently underway. These scientifically designed surveys are comparable with those undertaken in the UK and US providing important information to inform planning, shape policy and help with the implementation of service delivery [20]. However none of these surveys were specifically designed to monitor the impact of water fluoridation on oral health. Inferences can be drawn but larger samples are required to provide robust data on the effectiveness of water fluoridation in NSW.

However it is useful to compare the Statewide Child Survey (2007) dmft scores with those of the three study areas to note if there are any major differences. The overall mean dmft score for five year olds in the NSW state survey was 1.62, whilst the three study areas had dmft scores of 1.40 for Wyong (fluoridated) 2.02 for Gosford (pre-fluoridated) and North Coast 2.09 (non-fluoridated). Given that most children in the NSW Survey have access to fluoridated water, it is more appropriate to split the Statewide data according to fluoridation status, with the caveat that only a small proportion (15.4%) of the sample live in non-fluoridated areas. The Child Survey (2007) dmft mean scores are then 1.40 (fluoridated) and 2.62 (non-fluoridated). Wyong has the same dmft score of 1.40, whilst Gosford (PF) 2.02 and North Coast (NF) 2.09 are somewhat lower than the state score of 2.62, although the 95% confidence intervals for the non-fluoridated sample from the NSW Survey are fairly wide ranging, from 1.89-3.36. Fortunately the Area Health Service which includes Wyong and Gosford LGAs within its administrative area did take the opportunity to undertake a local survey with a larger sample size at the same time as the State Wide epidemiological exercise. Their 2007 results [21] reported a mean dmft for five year olds of 1.51 for the fluoridated areas and 2.03 for the non-fluoridated areas. These’s are close to the 2008 data presented in this paper and therefore on balance we consider the baseline dmft scores are an accurate reflection of the dental health of young children in the study areas. When Gosford City Council agreed to fluoridate, there was an opportunity to record the baseline levels of dental caries in 5-7year olds from both fluoridated and non-fluoridated areas of NSW. A survey of oral health with a sufficient sample size would enable future studies to monitor the oral health of children living in Gosford LGA over time, even though state based dental epidemiology is well developed. The three examination sites are all close to the NSW Coast, but the North Coast LGAs are smaller and more rural. Never the less they do offer useful comparison data to monitor the effect of fluoridation in Gosford LGA.

The lower recruitment rate of children into the study from Ballina and Byron LGAs was not unexpected as there has been considerable antagonism to water fluoridation, led in part by a group of local councilors. Hence some parents may have been reluctant to allow their children to join in the research project which was linked to water fluoridation. This may explain why four school principals refused to cooperate with the study.

NSW is a large state and the vast distances can impose logistical difficulties when undertaking research projects in a number of sites. The Public Dental Service is however a coherent organisation and this project was managed by senior dental therapists who had participated in a number of previous epidemiological surveys. The Gold examiner had to undertake considerable travelling to monitor progress, and undertake duplicate examinations, however the local public dental staff delivered the survey on time and within a limited budget. The data were collected on mark sense cards because the move to computer based direct entry was not sufficiently well developed to risk problems in geographically distant sites. Future surveys will be direct entry on to lap top computers which will reduce the time required for data collection and checking.

The results certainly show the impact of water fluoridation in Wyong and it will be interesting to see how the caries rates change in Gosford now that the new scheme has been implemented. The current survey should at least show the people of Gosford how the dental health of their children could improve over the next few years [22].

## Conclusion

The levels of dental caries are significantly less in the fluoridated town of Wyong compared with Gosford which was about to fluoridate and the Shires of Ballina and Byron which at that time had not agreed to accept water fluoridation.
